# Neisseria oralis Spondylodiscitis: A Case Report and Review of the Literature

**DOI:** 10.7759/cureus.80152

**Published:** 2025-03-06

**Authors:** Laurence Bamps, Lokmane Taihi, Ahalieyah Anantharajah, Jean Cyr Yombi

**Affiliations:** 1 Internal Medicine and Infectious Diseases, Cliniques Universitaires Saint Luc, Brussels, BEL; 2 Immunity and Cancer Research Group, De Duve Institute, Université Catholique De Louvain, Brussels, BEL; 3 Medical Imaging, Cliniques Universitaires Saint Luc, Brussels, BEL; 4 Clinical Microbiology, Cliniques Universitaires Saint Luc, Brussels, BEL

**Keywords:** commensal neisseria spp, emerging pathogen, neisseria oralis, opportunistic pathogen, spinal osteomyelitis, spondylodiscitis

## Abstract

The pathogenicity and potential virulence of commensal *Neisseria *spp.* *are not fully understood, but growing literature describes cases of opportunistic infections in humans, including invasive or life-threatening presentations even in the absence of immunosuppression. We report the first case of spontaneous* Neisseria oralis* spondylodiscitis in an otherwise healthy immunocompetent adult, with no formally identified portal of entry. The infection was cured after six weeks of oral ciprofloxacin. *Neisseria oralis *is an emerging pathogen and can be involved in invasive infections even in immunocompetent individuals. Iterative changes in the taxonomy within the genus *Neisseria* over the years might have led to biases in the reporting of such cases.

## Introduction

Invasive infections caused by commensal *Neisseria* spp., usually harmless inhabitants of the human upper respiratory tract [1], are rare yet growingly described [2]. Recent advances in molecular characterization leading to recurring changes in the taxonomy within the genus *Neisseria* may add to the confusion and cause underreporting [1,3]. Spondylodiscitis is an osteoarticular infection that affects both the intervertebral disc and the two adjacent vertebrae. Its origin is most often bacterial, following transient or sustained bacteremia, due to the vascular configuration in the vertebral endplates, which favors septic emboli. In such cases, it is predominantly localized in the lumbar spine (58%). In a quarter of cases, no microbiological diagnosis will be established. Nevertheless, the list of causative microorganisms has been expanding over the years, including emerging pathogens [4]. We report here a rare case of* Neisseria oralis* spondylodiscitis in a non-immunocompromised patient and review the literature.

## Case presentation

A 64-year-old male consulted the outpatient clinic for debilitating lumbar pain, which slowly increased over the past nine weeks without any identified trigger or trauma. His past medical history was significant for a left pleurectomy due to spontaneous pneumothorax, and a transurethral resection of the prostate performed for benign prostate hyperplasia, several years prior to presentation. He did not have any history of lower back pain. The pain was refractory to medical management (paracetamol and diclofenac). He denied any fever or weight loss but mentioned mild flu-like symptoms at pain onset. He practiced proper oral hygiene, including daily use of a water flosser, his last visit to the dentist occurred 10 months ago for his yearly teeth scaling, and he did not report any oral or periodontal disease or injury, apart from the placement of dental implants more than five years ago. He did not report any illicit drug use. He took rabeprazole as the only chronic medication for gastroesophageal reflux.

**Figure 1:**
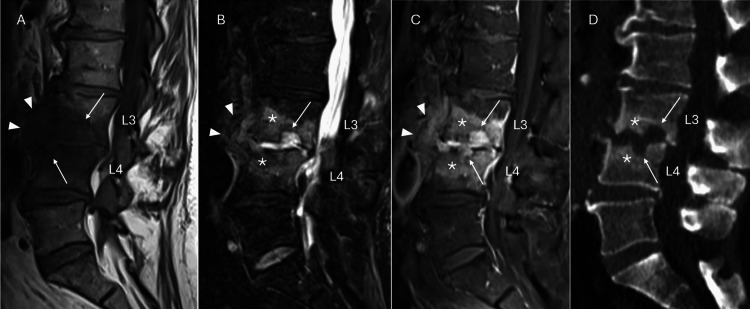
Sagittal lumbar spine sequences: A) MRI T1W, B) MRI T2W with fat suppression, C) MRI T1W with fat suppression and gadolinium injection, and D) sagittal reconstruction of lumbar spine CT scan. MRI showed bone marrow edema at L3-L4 (stars), adjacent to endplate erosions (arrows). The erosions and intervertebral space were filled with highly hydrated tissue (marked hyperintensity in T2W images [B]), which was highly enhanced after gadolinium injection (C), corresponding to granulation tissue, with prevertebral swelling and infiltration (head arrows). In the CT scan (D), the erosions were deep with blurred margins (arrows), associated with reactive sclerosis of the trabecular bone (stars). MRI, magnetic resonance imaging; T1W, T1-weighted; T2W, T2-weighted; CT, computed tomography

A first computed tomography (CT) scan was performed, which identified common degenerative changes in the lumbar spine (Figure 1). Due to ongoing pain, a magnetic resonance imaging (MRI) was performed one month later revealing erosive lesions of L3-L4 endplates with bone marrow edema and soft tissue infiltration, indicative of spondylodiscitis (Figure 1). No collection formation nor epidural extension were observed. He was referred to our internal medicine and infectious diseases ward for hospital management of spondylodiscitis.

Admission vital signs were a blood pressure of 142/90 mmHg, a heart rate of 78/min, oxygen saturation of 99% on room air, and an axillary temperature of 35.2°C. Physical examination revealed painful vertebral percussion around the L3 level, paravertebral muscle tenderness, and limitation of spine movement, without neurological defect. No suggestive signs of endocarditis, such as a heart murmur or septic embolism, were found. Laboratory tests revealed a C-reactive protein (CRP) level of 22.6 mg/L (normal range <5 mg/L), with no abnormalities in the white blood cell count; the rest of the biochemical analyses were unremarkable.

During the hospital stay, eight sets of aerobic and anaerobic blood cultures (BD BACTEC™; Becton, Dickinson and Company, New Jersey, USA) were collected, and all remained negative after five days of incubation. Serologic testing for Brucella and QuantiFERON®-TB Gold (Interferon Gamma Release Assay) was also negative. A whole-body 18F-fluorodeoxyglucose positron emission tomography-CT confirmed the image of L3-L4 spondylodiscitis and showed no other infectious foci or portal of entry. A transthoracic echocardiography showed no sign of endocarditis. Meanwhile, the patient remained afebrile, showing no change in his symptoms and clinical signs. CRP fluctuated within low to normal values (4 to 22.6 mg/L). To allow the identification of the causal pathogen, a percutaneous disco-vertebral needle biopsy was performed eight days after admission. Four tissue samples were received. For each sample, BD™ Columbia Agar supplemented with 5% sheep blood was inoculated under CO₂ conditions at 35°C, and BD™ Brucella Agar supplemented with 5% horse blood was inoculated under anaerobic conditions at 35°C. Histopathology confirmed the presence of a subacute inflammatory infiltrate of the osteochondral tissue, involving neutrophils along with focal necrosis. Nevertheless, gram staining on the samples was negative, and all cultures remained inconclusive after 10 days of stay. Because the patient remained clinically stable throughout his hospital stay, and to avoid compromising the possibility of additional sampling in case microbiological results were not obtained after the initial procedure, a decision was made to discharge the patient without starting antibiotic therapy while awaiting the results.

The day after he was discharged, i.e., 48-72 hours after incubation, *Neisseria oralis* grew on the Columbia medium under CO₂ conditions in all four samples. No other organisms were detected. The anaerobic medium remained negative. The species was identified using matrix-assisted laser desorption ionization time-of-flight mass spectrometry (MALDI-TOF MS), with a score of 2.23 and consistency category A (Microflex LT mass spectrometer and IVD MALDI Biotyper System, Bruker, Bremen, Germany; MALDI Biotyper (MBT) IVD Compass, software version 4.2.90; MBT IVD Library, DB8326, database version 9).

**Figure 2:**
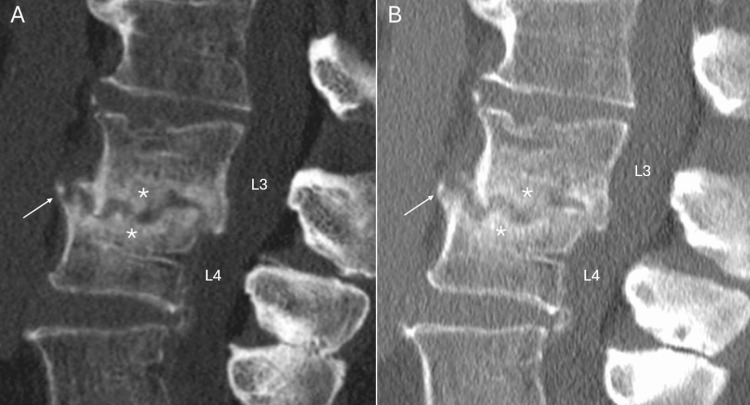
Sagittal reconstruction of lumbar spine CT scan, taken two months (A) and seven months (B) after the end of treatment. Follow-up CT scan showed bone reformation, seen as sclerosis of trabecular bone (stars) and osteophytosis (arrows), with no signs of progressive erosions, consistent with post-infectious ankylosis of L3-L4. CT, computed tomography

Antibiotic sensitivity testing (AST) reported sensitivity to ceftriaxone (minimum inhibitory concentration 0.064 mg/L) and ciprofloxacin (0.032 mg/L), and reduced susceptibility to penicillin G (2 mg/L) and rifampicin (2 mg/L). The patient was subsequently started on oral ciprofloxacin 750 mg b.i.d. and followed up at the outpatient clinic. He showed an improvement in his symptoms and sustained normalization of CRP. Ciprofloxacin was discontinued after a six-week course. A dental assessment was conducted after discharge, and no oral or dental pathology was detected. Two follow-up CT scans were performed (respectively two and seven months after the end of treatment) due to persistent lumbar pain, showing osteoarticular changes compatible with spondylodiscitis sequelae and a progressive evolution toward L3-L4 ankylosis (Figure 2), with no signs of residual active infection.

## Discussion

Among the genus *Neisseria*, *Neisseria gonorrhoeae* and *Neisseria meningitidis* are the main pathogenic species in humans (the first being an obligate pathogen, the second predominantly commensal but capable of major virulence) [5]. Non-meningococcal, non-gonococcal species of* Neisseria* are considered human commensals [1]; however, they have increasingly been reported as causative agents of invasive diseases, including life-threatening presentations such as endocarditis or meningitis [2,5]. Several mechanisms and risk factors have been suggested, including immunosuppression, recent oral mucosa trauma, poor oral hygiene, and intravenous drug use [2,5]. A recent review of 233 reported cases of commensal Neisseria infections showed that only 14.2% were predisposed by immunosuppression, while only 7.7% involved previously healthy patients [5]. However, the mechanisms of virulence of these species remain unclear [2].

*Neisseria oralis* sp. nov. was reported in 2013 as a novel species, phylogenetically close to *Neisseria lactamica* but forming a distinct clade according to 16S rRNA gene sequencing, based on seven isolates collected from the subgingival plaque of seven healthy American individuals [6]. Subsequent research compared this novel species to other members of the genus *Neisseria* using whole genome sequencing and concluded that *Neisseria* oralis is the same species as the previously named *Neisseria* mucosa var. *heidelbergensis* [3]. The species *Neisseria mucosa* has been divided since 1971 into two different varieties, including var. *heidelbergensis*, which produces a yellow pigment [7].

To our knowledge, since it was established as a distinct species, only two cases of invasive infections caused by *Neisseria oralis* were reported: one case of cystitis in a diabetic adult [8], and one neonatal sepsis with bacteremia [9]. Meanwhile, under its previous name, *Neisseria mucosa* var. *heidelbergensis* was reported in only one case of acute meningitis in a severely handicapped five-year-old girl [10]. Given that the name "*Neisseria mucosa*" (variant unspecified) has been used to designate different species throughout taxonomic revisions, it is not possible to determine which of the reports [2] in the literature published before 2013 [6] might have involved what is now called *Neisseria oralis*. Among these, only two cases of osteoarticular infections have been reported: one septic arthritis of the knee following an intra-articular injection in 1985 [11], and one spontaneous septic arthritis of the knee in a patient taking corticosteroids [12].

In our case, the patient’s medical history and work-up did not reveal any risk factors or obvious predisposing conditions. His dental implants were not considered the most likely causal factor, given their long-standing presence and the absence of signs of local complications; however, this hypothesis cannot be ruled out. The daily use of a water flosser, causing breakage of the dental plaque, might have potentialized transient bacteremia even with a good periodontal status [13]. The clinical presentation was slow and insidious, making the diagnosis challenging on one hand, but also setting this case report apart from most other infections caused by commensal *Neisseria*, which are predominantly described as acute, severe, or life-threatening [5].

The diagnosis was allowed by standard culture on disco-vertebral samples. As all the samples collected under sterile conditions grew the same species in pure culture, contamination is unlikely, despite the negative blood cultures. Identification of* Neisseria oralis* relied solely on MALDI-TOF MS, as the identification score was high (consistent with the manufacturer’s criteria, consistency category A indicates reliable identification, with matches scoring ≥1.9 being of the same species and matches scoring ≥1.7 being of the same genus). Our laboratory does not routinely use genome sequencing, though it would have been ideal to confirm the identification using this method, particularly given the rarity of reported cases of invasive infection with this species. Nevertheless, although several studies have described misidentifications of *Neisseria* spp. with MALDI-TOF MS, as recently reviewed by de Block et al. [14], their recent surveillance study conducted in isolates from a Belgian cohort showed that all *Neisseria oralis *isolates identified with MALDI-TOF, using the same database as ours, were accurately verified by ribosomal multilocus sequence typing.

The patient was treated with a full six-week course of oral ciprofloxacin, resulting in a sustained favorable outcome after seven months. However, longer-term follow-up will be even more informative, given the slow and insidious nature of the initial presentation.

## Conclusions

We report the first case of spondylodiscitis caused by *Neisseria oralis*, with the limitation of the absence of molecular confirmation for strain identification, in a non-immunocompromised adult male, with no formally identified portal of entry or associated bacteremia.

Although iterative changes in taxonomy may lead to bias in under- or misreporting, only two cases of osteoarticular infection (none involving the spine) have been reported with *Neisseria mucosa*, a species in which *Neisseria oralis* was historically misclassified. To our knowledge, this is the first reported infection involving *Neisseria oralis* at an osteoarticular site.

## References

[1] Bennett JS, Jolley KA, Earle SG, Corton C, Bentley SD, Parkhill J, Maiden MC (2012). A genomic approach to bacterial taxonomy: an examination and proposed reclassification of species within the genus Neisseria. Microbiology (Reading).

[2] Humbert MV, Christodoulides M (2019). Atypical, yet not infrequent, infections with Neisseria species. Pathogens.

[3] Bennett JS, Jolley KA, Maiden MC (2013). Genome sequence analyses show that Neisseria oralis is the same species as 'Neisseria mucosa var. heidelbergensis'. Int J Syst Evol Microbiol.

[4] Piccolo CL, Villanacci A, Di Stefano F (2024). Spondylodiscitis and its mimickers: a pictorial review. Biomedicines.

[5] Walsh L, Clark SA, Derrick JP, Borrow R (2023). Beyond the usual suspects: reviewing infections caused by typically-commensal Neisseria species. J Infect.

[6] Wolfgang WJ, Passaretti TV, Jose R (2013). Neisseria oralis sp. nov., isolated from healthy gingival plaque and clinical samples. Int J Syst Evol Microbiol.

[7] Berger U (1971). [Neisseria mucosa var. heidelbergensis]. Z Med Mikrobiol Immunol.

[8] Alamri Y, Keene A, Pithie A (2017). Acute cystitis caused by commensal Neisseria oralis: a case report and review of the literature. Infect Disord Drug Targets.

[9] Baniulyte G, Svirpliene S, Eccleston A, Arjunan S, Connor M (2021). Neisseria oralis septicaemia in a newborn: first recorded case. Paediatr Int Child Health.

[10] Berger U, Aboulkhair I, Rottmann W (1974). Septicaemia and meningitis caused by Neisseria mucosa varietas heidelbergensis. Infection.

[11] Abiteboul M, Mazieres B, Causse B, Moatti N, Arlet J (1985). Septic arthritis of the knee due to Neisseria mucosa. Clin Rheumatol.

[12] Van Linthoudt D, Modde H, Ott H (1987). Neisseria mucosa septic arthritis. Br J Rheumatol.

[13] Crasta K, Daly CG, Mitchell D, Curtis B, Stewart D, Heitz-Mayfield LJ (2009). Bacteraemia due to dental flossing. J Clin Periodontol.

[14] de Block T, De Baetselier I, Van den Bossche D (2024). Genomic oropharyngeal Neisseria surveillance detects MALDI-TOF MS species misidentifications and reveals a novel Neisseria cinerea clade. J Med Microbiol.

